# Ewing's Sarcoma of Rib in a Four Year Old: A Case Report

**DOI:** 10.31729/jnma.6481

**Published:** 2021-10-31

**Authors:** Saru Kunwar, Nisha Sharma, Bishnu Rath Giri, BC Bom, Anil Kumar Shrestha

**Affiliations:** 1 Department of Paediatrics, Kanti Children's Hospital, Maharajgunj, Kathmandu, Nepal; 2 Hemato-oncology Department, Kanti Children's Hospital, Maharajgunj, Kathmandu, Nepal; 3 Department of Radiology, Rapti Academy of Health Sciences, Ghorahi, Dang, Nepal; 4 Department of Paediatrics, Kanti Children's Hospital, Maharajgunj, Kathmandu, Nepal

**Keywords:** *Ewing's sarcoma*, *pneumonia*, *ribs*

## Abstract

Although rare, the Ewing's sarcoma family of tumors represents the second most common primary bone malignancy that typically occurs in adolescents and young adults aged 10-25 years, presenting with localized pain and swelling of a long bone. We report a case of Ewing's sarcoma of rib in a four years old child that presented with acute onset of fever, cough, shortness of breath and was initially treated as pneumonia. Although the patient did not belong to typical demography and the symptoms were suggestive of infective pathology, careful scrutiny of the radiographic findings led to further investigations and diagnosis of Ewing's sarcoma of rib after correlating computerized tomography scan findings with immunohistochemistry reports.

## INTRODUCTION

Ewing's sarcoma is the second most common primary bone malignancy. It constitutes 1 % of all cancers in children less than 15 years and 2% in teenagers aged 15 to 19.^1^ The most common sites of origin are the long bones of extremities and pelvic bones while ribs make up 10 % of the incidence. Localized pain and swelling are the most common presenting symptoms.^2^ The five year survival rate of Ewing's sarcoma is 75% for children younger than 15 and 58% for teens aged 15 to 19.^1^

We here discuss a case of a four years old child with Ewing's sarcoma of right 8th rib whose initial clinical presentation resembled that of pneumonia.

## CASE REPORT

**Figure 1a, 1b, 1c:**
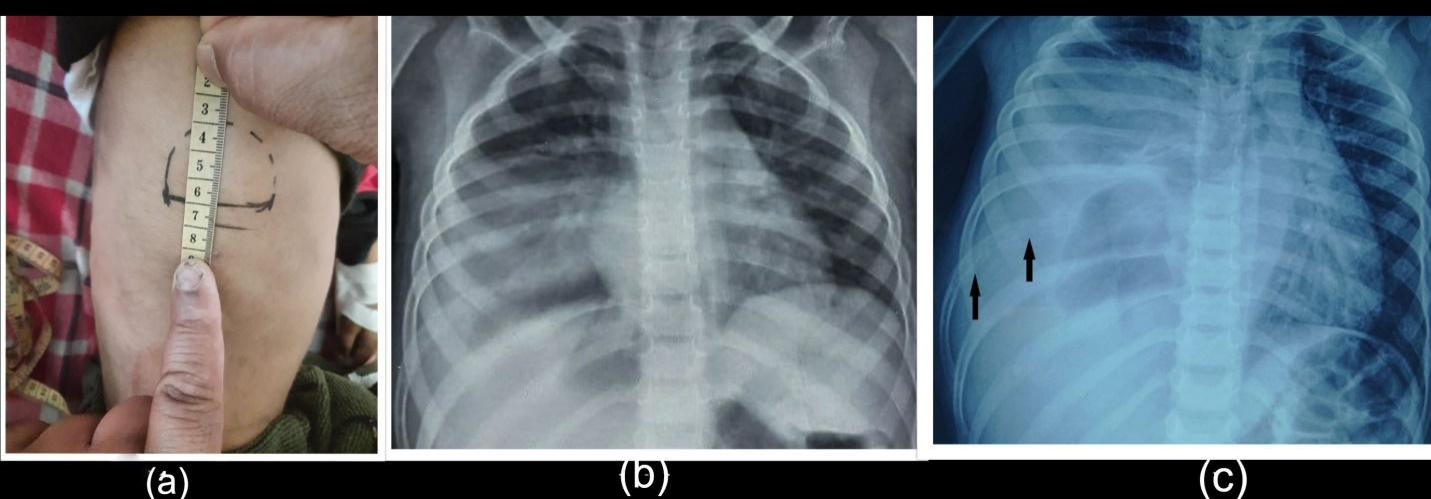
(a) Swelling over right lateral chest wall; (b) X-ray one month back (at initial presentation) to local hospital; (c) at the time of visit to our hospital.

A four years old male was brought to our emergency with chief complaints of acute onset fever (maximum temperature 38.9°C), non-productive cough and difficulty breathing for one and half months. He was admitted to a local hospital one month back with a diagnosis of pneumonia with pleural effusion where he had completed a seven days course of intravenous antibiotics. There was partial improvement of symptoms but it recurred after 4-5 days. Child was then prescribed oral antibiotics for one week. However, there was no resolution of symptoms and was hence brought to our center. Upon detailed assessment, there was a history of hard, painless swelling on the right side of his chest initially noticed six months back (Figure 1a) and a weight loss of three kg. His family history was nonsignificant. His respiratory rate at the time of admission was 30/min, SpO_2_ 96 % of room air and temperature was 36.8°C. On examination, he had decreased air entry on the right side and a palpable swelling 3x4.5 cm, hard, immobile, non-tender, round with smooth surface and ill-defined margins was noted on the right mid-axillary line in the lower chest wall (Figure 1a). His initial chest X-ray during his admission in local hospital one month back, showed right middle zone and lower zone radio-opacity (Figure 1b). However, chest x-ray done at our hospital showed homogenous radio-opacity involving the middle and lower zones of the right lung along with mediastinal shift to the left side (Figure 1c). On radiological evaluation, diffuse decrease in normal density of 8th rib suggesting permeative destruction was revealed.

**Figure 2:**
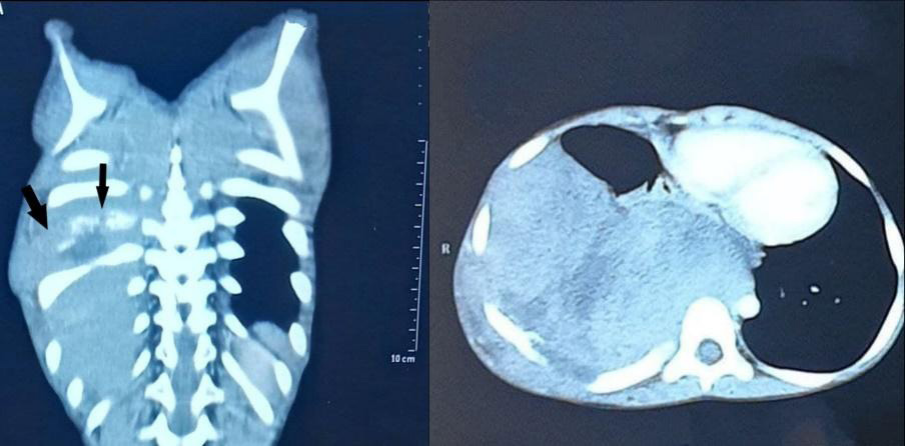
CT Chest showing lobulated heterogeneously enhancing mass in the right pleural cavity with posterolateral chest wall invasion and encasement of the right 8th rib which showed permeative erosion and expansion.

His WBC count was 7,000/mm^3^ with polymorphs 58%, lymphocytes 39%, and monocytes 3%; platelets count was 410,000/mm^3^, hemoglobin was 11.4 gm%, erythrocyte sedimentation rate 46 mm/1st hour and C-reactive protein was positive. Patient was then admitted to pediatrics inpatient department and ultrasonography of the chest was done which revealed a heterogeneous area measuring approximately 11x8.8 cm in the right lung with minimum vascularity within it and no significant effusion. Contrast enhanced CT scan of the chest was done that showed lobulated heterogeneously enhancing mass in the right pleural cavity with posterolateral chest wall invasion. The lesion had encased the right 8th rib with permeative erosion and expansion along with a very minimal amount of free fluid in the right pleural cavity. There was also passive collapse and consolidation of the right middle lobe and basal segments of right lower lobe with a contralateral shift of mediastinum. CT diagnosis of pleuro-pulmonary blastoma with chest wall invasion was given with Ewing's Sarcoma of right 8th rib (Figure 2) as a differential diagnosis.

Serum lactate dehydrogenase was raised with a value of 1370 U/L; however, serum beta-HCG and AFP values were within normal range. 99M TC-MDP whole body bone scan revealed increased uptake in the right 7-9th ribs. Biopsy of the chest wall lesion was carried out. On microscopy, a poorly differentiated malignant tumor, likely a malignant small round cell tumor was noted. The immunohistochemistry findings were consistent with Ewing's sarcoma (Table 1).

**Table 1:** Immunohistochemistry report consistent with Ewing's Sarcoma.

IHC Markers	Results
CD 45	Non-immunoreactive, score 0 in neoplastic cells
Vimentin	Immunoreactive, score 4+ in neoplastic cells
CD 99	Immunoreactive, score 4+ in neoplastic cells
Desmin	Non-immunoreactive, score 0 in neoplastic cells
FLI-1	Immunoreactive, score 3+ in neoplastic cells
Ki-67	Immunoreactive in 50-60% of neoplastic cells

**Figure 3:**
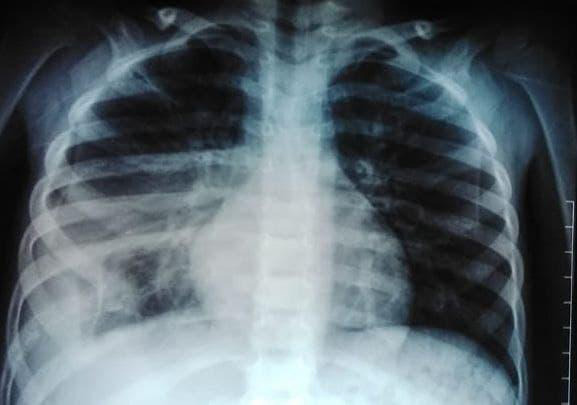
Chest X-ray after two cycles of chemotherapy.

With above histological, immunohistochemistry and radiological findings, final diagnosis of Ewing's sarcoma of 8th rib was reached and the patient is currently on intensive multidrug induction chemotherapy regimen containing vincristine, ifosfamide, doxorubicin and etoposide (VIDE) along with Mesna. Chest X-ray was done after two cycles of chemotherapy and it showed significant decrement in radiopacity (Figure 3).

The Fluorescence in-situ Hybridization (FISH) for Ewing's Sarcoma (EWS), 22q12 (EWSR1) rearrangement was negative and his bilateral bone marrow biopsy report was negative for malignant cells.

## DISCUSSION

The most common presentations of Ewing's sarcoma are local pain and swelling. Systemic features like fever, weight loss, anemia, leukocytosis and an increase in ESR are not frequently seen unless the disease is quite advanced.^3^ Ewing's Sarcoma of the ribs can manifest as an extra-pleural mass since it has propensity to spread inwards towards the thoracic cavity. Approximately 20-25% of patients present with metastases at diagnosis.^4^

Initial investigation with X-Ray is helpful and cost effective. It usually shows typical multiple, confluent, lytic bone lesions giving rise to images described as “moth eaten" and sub-periosteal growth revealing two classic images - Codman's triangle and the “onion peel" appearance. 5 While assessing a chest X-ray of child presenting with respiratory symptoms there is usual tendency of considering pneumonia with pleural effusion as the only diagnosis since it is a more common disease among children. However, similar to the present case, there have been other cases in literature where failure to look at all aspects of X-ray has led to misdiagnosis/delay in diagnosis and management even though the lesion seemed obvious.^3,9-13^

Translocation t(11, 22) (q24; q12) is pathognomonic for Ewing's sarcoma, occurring in 85% of patients and it gives rise to the formation of the EWS-FLI 1 fusion gene.7 (EWS), 22q12 (EWSR1) rearrangement was negative in the present case. However, diagnosis of Ewing's Sarcoma should not be ruled out in the absence of molecular confirmation but prompt review of clinical, histological, and immunohistochemical features should be done.^8^

A chest CT scan is usually done to rule out metastasis to lung or pleura. The screening for other bone metastases consists of 99mTc bone scintigraphy. The fluorodeoxyglucose positron emission tomography (FDG-PET) scan is also useful for staging, restaging, and assessing response to chemotherapy but the downside is that it is expensive and not readily available in developing countries like ours.

Histologic examination of Ewing's sarcoma typically reveals sheets of small, round, blue cells with a prominent nucleus and scant cytoplasm.^5^ Immunohistochemical findings, although not specific, include strong membrane expression of CD99 (also called MIC2), along with frequent expression of CD56 and synaptophysin; the absence of CD99 expression virtually rules out the diagnosis of Ewing's sarcoma.^14^’^15^

Ewing's sarcoma of the chest wall along with pleural involvement is rare. However, when a child is brought with symptoms of respiratory tract infection that doesn't respond well to antibiotics, evaluation and work up for Ewing's sarcoma or other childhood malignancy of thorax should also be considered.
